# In vivo effects of Pain Relieving Plaster on closed soft tissue injury in rabbit ears

**DOI:** 10.1186/1472-6882-8-51

**Published:** 2008-09-15

**Authors:** Yong-Zhi Wang, Chun-Yu Guo, Hong-Gang Zhong, Wan-Nian Zhang, De-Long Wang, Xuan Wang, Fu-Hui Dong

**Affiliations:** 1 Wangjing Hospital of China Academy of Chinese Medical Sciences, Beijing, 100102, PR China; 2 Xiyuan Hospital of China Academy of Chinese Medical Sciences, Beijing, 100091, PR China; 3 Institute of Orthopaedics and Traumatology, China Academy of Chinese Medical Sciences, Beijing, 100700, PR China

## Abstract

**Background:**

Soft tissue injury imposes major public health burdens worldwide. The positive effect of China's Tibetan medicine and the *Lamiophlomis rotata*-based herbal Pain Relieving Plaster (PRP) on healing closed soft tissue injury (CSTI) has been reported. The herbs contained in Plaster are also referred as 'blood-activating and stasis-dispelling' in herbal medicine. The formula of the plaster contains four China's Tibetan medical herbs, including *Lamiophlomis rotata*, *Oxytropis falcate Bunge*, *Curcuma longa Linn*, and *Myricaria bracteata*. Two of these herbs (*Lamiophlomis rotate*; *Curcuma longa Linn*) are commonly used in different formulae of Chinese medicine. The objective of this study is to use an interdisciplinary approach to test the hypothesis that the formula and its components influence the process of CSTI.

**Methods:**

In vivo models have been established in 30 rabbit ear pinnae and studied for: (1) blood flow velocity (BFV) which was affected by pressure of 21.2 kg/cm2 for 30 second over the local rabbit ear tissue; (2) edema formation of the closed soft tissue injury; (3) in vivo local temperature change.

**Results:**

The results of in vivo studies indicated that CSTI significantly increased the velocity of blood flow and increased edema formation within the control group. The PRP extracts for 5 hours significantly slowed down the BFV of CSTI in rabbit ears, markedly decreased the elevated edema level from the 3rd to the 5th day.

**Conclusion:**

The ingredients contained in the formula have positive effects in healing CSTI and further study is worth exploring.

## Background

The overwhelming majority of injuries caused by a variety of activities as well as high-velocity accidents typically involve moderate to severe trauma to the musculoskeletal soft tissues. Clinical and investigational experience has shown that primary traumatic muscle injury induces a plethora of pathological alterations known to converge in secondary structural and functional deterioration [1]. This secondary lesion growth is related to progressive microcirculatory impairment characterized by endothelial damage, local activation of the coagulation cascade, and marked leukocyte recruitment leading to, among others, decreased nutritive blood flow, reduced oxygen delivery, sustained cellular metabolism, and production of autodestructive free oxygen radicals [2].

While the primary insult cannot be influenced therapeutically, the secondary lesion growth after CSTI is amenable to certain interventions, including temporary immobilization, administration of analgesic anti-inflammatory drugs, and some traditional herbs such as China's Tibetan Medicine [3-6]. The World Health Organization estimated that about 80% of the world's population still relies on plant-based medicines for their primary health care. 7 This in fact is a clear indication for the role of medicinal plants in the maintenance of health and treatment of diseases as therapeutic alternatives throughout the world, still in the late 20th and early 21st century [7]. Soft tissue injury care can be traced back to early civilizations, and many of these treatments were based on the use of herbal remedies. Approximately one-third of all traditional medicines in use are for the treatment of wounds and soft tissue disorders, compared to only 1%–3% of modern drugs [8].

Many traditional remedies are based on systematic observations and methodologies and have been time-tested, however many of them are still lacking scientific evidence. There are only a few prospective randomized, controlled trials that have proved the clinical efficacy of these traditional healing agents for soft tissue injury. Pain Relieving Plaster (PRP) composed of herbs only from China Tibetan Plateau, most of which have been used clinically for hundreds of years in China to remove stasis and relieve pain [9,10]. The present study was designed to test the in vivo CSTI healing activity of the extracts of PRP, namely:*Lamiophlomis rotata*, *Oxytropis falcate Bunge*, *Curcuma longa Linn*, and *Myricaria bracteata*.

## Methods

### Composition and Preparation of PRP

*Lamiophlomis rotata*,*Curcuma longa Linn*, *Oxytropis falcate Bunge *and *Myricaria bracteata *are the main composition. PRP is produced by the Tibet Linzhi Cheezheng Tibetan Medicine Factory. The composition of PRP is listed in Table 1. The PRP was extracted and evaporated to obtain a concentration equivalent to 0.5 g/mL crude drug for administration.

**Table 1:** HPLC screening of extracts from four plants of PRP

Plant name	Constituents				
	Flavone	Flavonoids	Alkaloids	Curcumin	Volatile oils

Lamiophlomis rotata	+	++	-	-	+
Oxytropis falcate Bunge	++	+	+	-	+
Curcuma longa Linn	-	-	+	++	+
Myricaria bracteata	+	-	-	-	-

(-) not detected; (+) positive; (++) strongly positive

### Analyses the Maker Components in PRP

Flavonoids, Curcumin, and Flavone were the maker components of *Lamiophlomis rotata*, *Curcuma longa Linn*, *Oxytropis falcate Bunge *and *Myricaria bracteata *(Table 1). These compounds were analyzed by high performance liquid chromatography (HPLC) compared with standard. A high-pressure pump (L-7100, Hitachi, Tokyo, Japan) with interface (D-7000) was connected to a column (Mightysil RP-18, GP 250 × 4.6 mm) and an ultraviolet detector (Hitachi L-7420). Fine powder was dissolved in methanol and shaken at 200 rpm for 60 min. The suspension was then centrifuged in 6000 rpm for 5 min to separate the supernatant. Flavonoids, Curcumin, and Flavone were detected at UV spectra of 320 nm, 280 nm, and 203 nm, respectively.

### Animals

White New Zealand rabbits of mean weight 2.5 ± 0.1 kg were supplied by and kept at The Laboratory Animal Services Centre, China Academy of Chinese Medical Sciences (CACMS). Animals were kept for one week to be acclimatized prior to the investigation. During this time they were given standard diet and water. All animals were housed, fed and treated in accordance with the in-house guidelines for animal protection. The study complied with the Declaration of Helsinki, and the study protocol was approved by the institutional ethics committee.

### Closed Soft Tissue Injury induction

Twenty four hours before the beginning of the modeling, the dorsal skin of the rabbit ears were shaved and the deep hair were removed by barium sulfide suspension. In the next day and under phenobarbital sodium anesthesia (1 ml/kg), a 6 mm × 6 mm injury was created by applying a soft tissue clamp with pressure of 21.2 kg/cm2 controlled by mechanical sensor for 30 seconds over the rabbit ear tissue.

### Experimental protocol

After the CSTI to the rabbits, ears were randomly subjected to surface application for 30 minutes with herbal extracts (PRP) or 0.9% NaCl (controls; room temperature) (n = 10 per group). Rabbits remained anesthetized for 5 hours during which determination of intravital microscopy, edema formation and temperature change were conducted. Uninjured rabbits subjected to the same procedures served as shams (n = 10), considering the effects that different steps may bring.

### Microcirculatory Analysis

Quantification of CSTI microcirculation and microvascular parameters was performed by the same investigator, who was blinded to the status of the rabbits. The ear was focused with the objective lens of the microscope on some spot just beneath the injury being examined. The image of the examined capillaries and the red cells inside them can be viewed with the eyepiece of the microscope(XSZ-HS7, Sony, Japan) and a digital video camera (DV, type: SONY TRV900) simultaneously. The images of the venules and capillaries and the red cells were projected on the charge coupled device (CCD:GP410, Panasonic, Japan) connected to an video recorder (PM-1442QM, SONY, Japan) and they can be recorded on videotape for analysis at a later time with image processing techniques (MV300, AverMedia, Taiwan) via computer [11]. Videotaped sequences were evaluated by a frame-to-frame analysis using a computer-assisted image analysis program for the BFV in capillaries and venules. The BFV was measured from a line shift diagram by drawing a line along the length of the center of the vessel and assessing the vertically aligned pixels under this line during a 10-second observation period [12,13]. The velocity was measured at the time of 0.5, 3, and 5 hours after CSTI respectively.

### Determination of soft tissue edema formation and temperature change

The soft tissue edema formation is part of the injury effect. A caliper for measuring soft materials (DT-320, Tianjin, China) was used to evaluate successive change of edema formation. The edema thickness gain was assessed by measuring the difference that remains after the basic thickness was subtracted from the posterior one. This differs from the wet-to-dry weight ratio previously described by others [14,15]. In order to get repeatable, quantitative, easily obtainable edema formation data in temporal sequence at one rabbit ear, it was measured by the caliper within 0.5, 3, 5 hours and in the successive 1 to 5 days. Temperature change was measured by infrared temperature tester (CH-13, Jiangsu, China).

### Statistical Analysis

Results are expressed as mean ± SD. The groups were compared using repeated – measures ANOVA(SPSS, Version 12.0 for Windows, SPSS Inc., Chicago, IL, USA). The results from different time points were analyzed by group t-test to test differences from groups. Differences were considered significant at p < 0.05.

## Results

### CSTI area microcirculation

**Figure 1:**
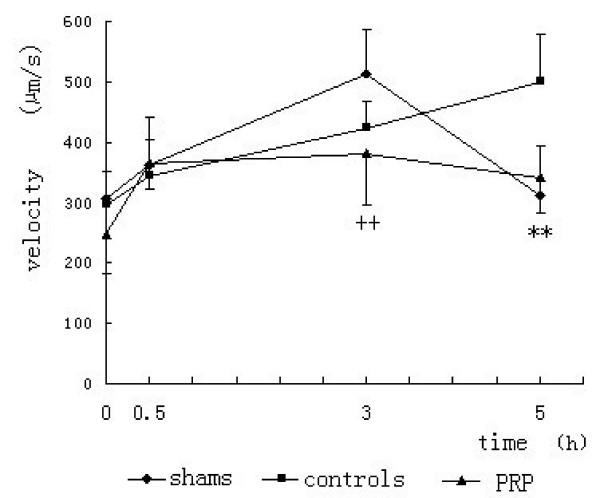
**Effect of PRP extracts on different groups**. Asterisks and the symbol (+) between points indicate the results of Tukey post hoc tests comparing means for the groups (*p < 0.05; **p < 0.01). No other between group comparisons indicated a statistically significant difference for the groups.

**Figure 2:**
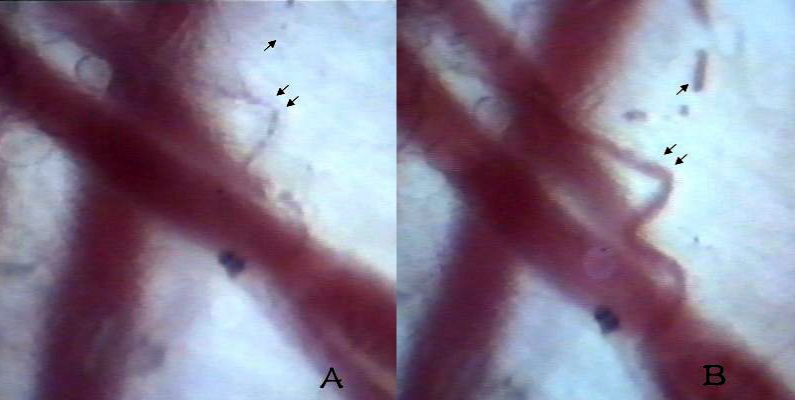
**Comparison of photographs at different hours**. A comparison of photographs documenting CSTI in the microcirculation of the rabbit ear treated with PRP extracts, in the baseline conditions (A), and within 5 hours (B) of reperfusion. Post-CSTI microvascular perfusion stopped in some microvessels (A, arrow). Blood flow decreased significantly with the evidence of the increased capillary diameter (B, arrow) and the reflow phenomenon (B, double arrow). (×640).

The results of in vivo studies indicated CSTI increased the BFV significantly. The PRP extracts at the 5th hour significantly slowed down the velocity compared with the controls, and the extracts could speed up the BFV of normal tissue (Figure 1; Figure 2). No interaction of time × group difference was found.

### Edema formation

**Figure 3:**
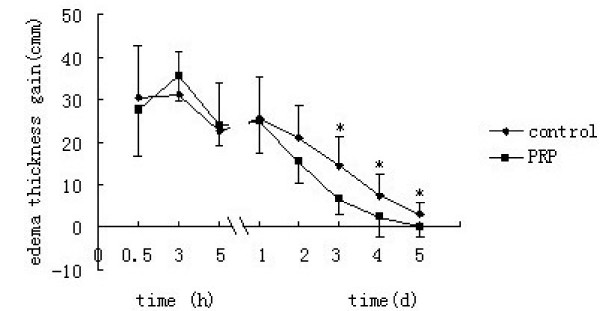
**Edema gain after two days**. No significant difference within 5 hours. A significant decrease in edema gain after two days (*p < 0.05).

The caliper for soft materials can easily measure the edema formation induced by CSTI in temporal sequence, as well as the reducing edema due to PRP treatment. No significant difference between the edema gain of the control group and the PRP group was observed within 5 hours. However the PRP application for half hours caused a significant decrease in edema gain after two days (Figure 3). There is no interaction of time × group.

### Ear pinna surface temperature

**Figure 4:**
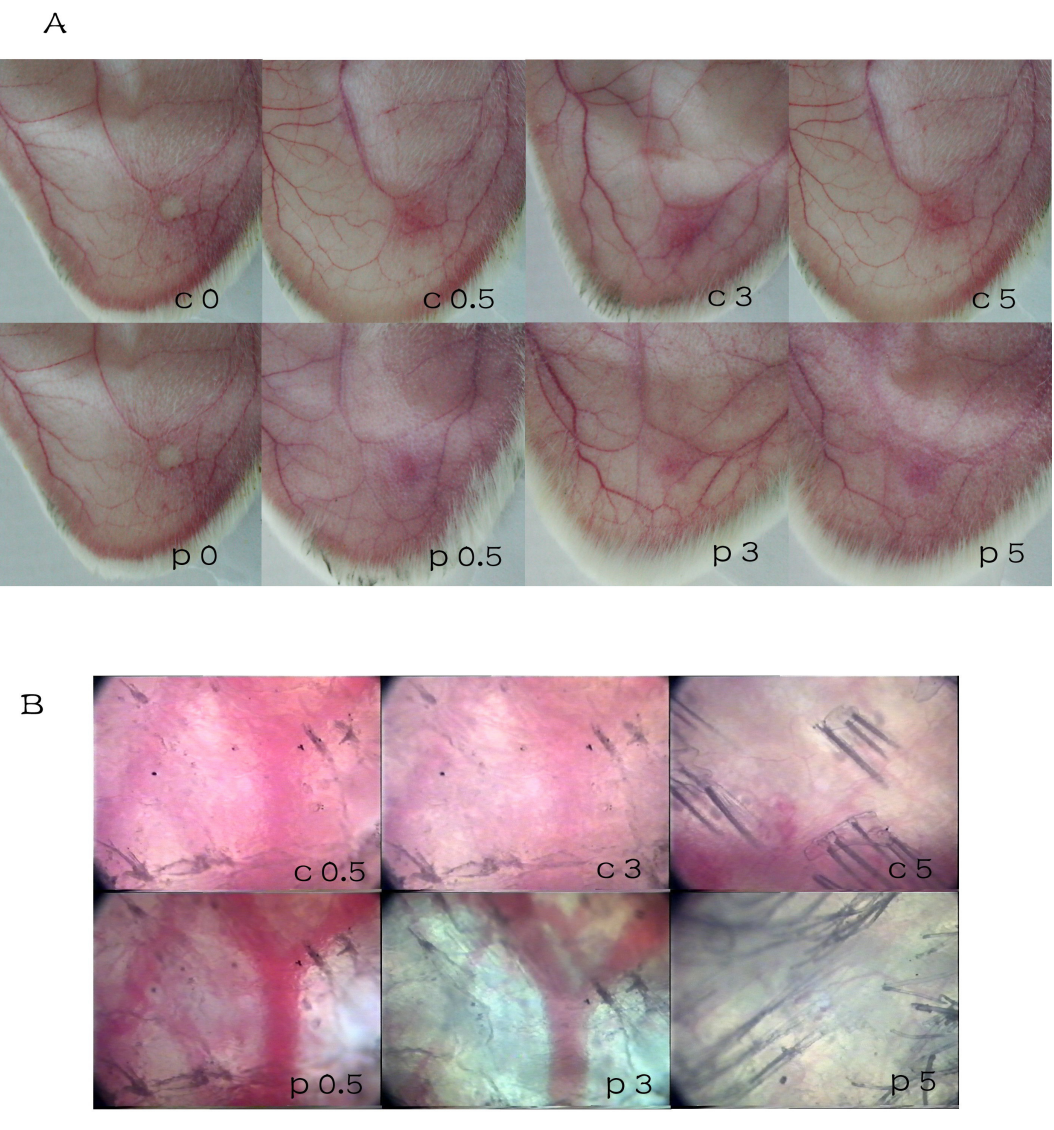
**CSTI area photographed at different hours**. Rabbit ears CSTI area photographed at different hours (0, 0.5, 3 and 5 hours). Macroscopically visible increase of CSTI area swelling and redness in control group as compared with PRP group before and after application of 0.9% NaCL (C0–C5: controls) or PRP extracts (P0–P5: PRP) (A), and the photograph under microscope (B)(×320).

There is no significant difference between the temperature of control group and PRP group (Table 2), though we find the CSTI area flushing differently within 5 hours (Figure 4).

**Table 2:** Temperature change at different time (°C)

Group	n	0 h	0.5 h	3 h	5 h
Control	10	30.16 ± 2.04	30.37 ± 2.67	32.18 ± 2.29	31.41 ± 2.01
PRP	10	29.71 ± 2.36	29.23 ± 3.20	31.65 ± 1.86	30.8 ± 3.27

No significant difference between the temperature of control group and PRP group.

## Discussion

In this study, we found that PRP extracts could significantly prevent the deterioration of CSTI and decrease the edema formation in rabbit ears. The results suggested that PRP has active medical ingredients for activating the blood and removing stasis. We also found that the application of PRP extracts could decrease redness in rabbit ears which is one of the signs of inflammation.

Significant increase in BFV in short time after injury suggests that decreased tissue oxygenation is essential to support and maintain cellular viability and impairment known to compromise structural and functional cellular integrity [16,17]. Both the blood flow and the microcirculation of the CSTI area were found significantly increased immediately after the injury in the control group, compared to the pre-injury values and that of the PRP group. The PRP extracts applied to the local injury significantly reduced the abnormal BFV induced by process of CSTI. In the sham group, blood flow and microcirculation remained elevated only in the 3rd hour indicating PRP could speed up BFV of normal tissue in rabbit ears in short time. The images from the camera and vital microscopy suggest that the blood flow is subjected to more congestion in the control group because of the abnormal flow pattern seen, and possibly because of a superior inflow or injury status found in these ears. A to-and-fro phenomenon of blood flow was noted in both groups followed by a reversed direction of flow in part of the microvasculature. These findings may be explained partially by the normal physiologic pressure gradients present in the microvasculature of the tissue [18].

The temperature result suggested that CSTI-induced ear flushing and unchanged temperature could be mediated by peripheral activation of some other receptors and affected by the environment factors [19,20]. A growing number of reports concluded that ear temperature measurement is unreliable and by implication so is the device. But the rich vascularization, innervation and variations in skin properties (thickness, oil secretion and hair) do affect the temperature reliability to some extent [21]. Therefore, further study regarding to the temperature is required.

PRP is a combined herbal medicine of four herbs from the Tibetan Plateau in China. Individual herbs play roles in promoting the microcirculation, decreasing edema and relieving pain. *Lamiophlomis rotata *may improve *blood *circulation and remove stasis through inhibition of thrombin and activation of plasminogen [22]. In addition, the aqueous extract of *Lamiophlomis Rotata *orally administered in rats can increase the contents of fibrinogen and shorten thrombin time. 10 Among these effects, the significant geographic structure of *Lamiophlomis rotata *may be one of the reasons affecting the process of CSTI [10]. *Oxytropis falcate Bunge *with the anti-inflammation effect may decrease edema formation for many soft tissues [23-25]. *Curcuma longa Linn *and *Myricaria bracteata *have the effects of activating the blood and removing stasis, and relieve pain as well [26-28]. Furthermore, *Curcuma longa Linn *demonstrated anti-inflammatory activity. It may exert its anti-inflammatory activity by inhibition of a number of different molecules that play a role in inflammation [29]. Reports suggested that Tibetan plant species which have higher nitrogen concentrations and photosynthetic capacities compared with a global dataset could be one of the effects [30,5].

Prescriptions for Chinese medicine usually combine several herbs or other ingredients to gain the maximal therapeutic effect. According to data from the present study, PRP extracts possesses blood-activating and stasis-dispelling properties that are consistent with studies of each individual herb [31]. PRP extracts may have blood activating, and edema decreasing effects in patients who have CSTI and similar injuries.

## Conclusion

This study showed that PRP extracts could prevent the deterioration of CSTI and speed up BFV of normal tissue in rabbit ears. PRP is a crudely combined herbal medicine that contains many chemical compounds and unknown ingredients. Isolating and identifying the main active components of PRP and their mechanisms of action exceeds the settings of the present investigation and merits further study.

## Competing interests

The authors declare that they have no competing interests.

## Authors' contributions

FHD, HGZ participated in the design of the study and WNZ, DLW and CYG performed the statistical analysis. XW collected and interpreted the data. YZW, WNZ and HGZ carried out the studies and drafted the manuscript. All authors read and approved the final manuscript.

## Pre-publication history

The pre-publication history for this paper can be accessed here:


